# The Ly6 Protein Coiled Is Required for Septate Junction and Blood Brain Barrier Organisation in Drosophila

**DOI:** 10.1371/journal.pone.0017763

**Published:** 2011-03-15

**Authors:** Assia Hijazi, Marc Haenlin, Lucas Waltzer, Fernando Roch

**Affiliations:** 1 Université de Toulouse, UPS, Centre de Biologie du Développement, Université Paul Sabatier, Toulouse, France; 2 Centre National de la Recherche Scientifique, UMR 5547, Centre de Biologie du Développement, Toulouse, France; Universidade Federal do Rio de Janeiro, Brazil

## Abstract

**Background:**

Genetic analysis of the *Drosophila* septate junctions has greatly contributed to our understanding of the mechanisms controlling the assembly of these adhesion structures, which bear strong similarities with the vertebrate tight junctions and the paranodal septate junctions. These adhesion complexes share conserved molecular components and have a common function: the formation of paracellular barriers restraining the diffusion of solutes through epithelial and glial envelopes.

**Methodology/Principal Findings:**

In this work we characterise the function of the *Drosophila cold* gene, that codes for a protein belonging to the Ly6 superfamily of extracellular ligands. Analysis of *cold* mutants shows that this gene is specifically required for the organisation of the septate junctions in epithelial tissues and in the nervous system, where its contribution is essential for the maintenance of the blood-brain barrier. We show that *cold* acts in a cell autonomous way, and we present evidence indicating that this protein could act as a septate junction component.

**Conclusion/Significance:**

We discuss the specific roles of *cold* and three other *Drosophila* members of the Ly6 superfamily that have been shown to participate in a non-redundant way in the process of septate junction assembly. We propose that vertebrate Ly6 proteins could fulfill analogous roles in tight junctions and/or paranodal septate junctions.

## Introduction

The proteins of the Ly6 superfamily are an ancient feature of metazoan genomes, as genes coding for the Ly6 motif have been identified in a wide variety of animal clades, ranging from cnidarians [1] to vertebrates [2], [3]. The Ly6 domains are small extracellular modules of about 100 residues characterised by presence of 4–6 pairs of cysteines placed in stereotypical positions [4]. These conserved residues form internal disulphide bridges that stabilise the conformation of the motif, but the rest of the protein sequence can vary to a remarkable extent. Despite this variability, these proteins adopt upon folding comparable three-dimensional structures, that are characterised by an internal hydrophobic core supporting three protruding fingers [4]. Indeed, these architectural motifs are often referred to as Three Finger Domains (TFD). The Ly6 module is present in both soluble and GPI anchored membrane proteins but is never observed in combination with other extracellular motifs. Due to its plasticity, it has been co-opted into many different biological processes, where it participates as a protein-protein interaction domain binding specifically to a wide variety of molecular partners [5], [6].

The *Drosophila* genome codes for 45 proteins belonging to the Ly6 superfamily [7]. Further illustrating the versatility of the Ly6 module, three of these genes have been analysed at a functional level and have been found to participate in distinct developmental tasks, namely the assembly of the chitin extracellular matrix (*retroactive*) [8], the regulation of circadian rhythms (*sleepless*) [9] and the organisation of cell adhesion junctions (*boudin*) [7]. Thus, *Drosophila* represents an attractive system where to pursue genetic studies identifying the multiple physiological roles of these proteins.

We have analysed the role of another member of the fly Ly6 superfamily, the gene *CG2813/coiled* (*cold*) [10]. We show that *cold* mutants display similar phenotypes to those seen in *bou* alleles [7], indicating that *cold* is essential for the organisation of the insect septate junctions (SJ). These invertebrate adhesion structures contribute both to the maintenance of cell contacts and the establishment of paracellular barriers preventing the unregulated passage of ions and solutes through epithelial layers and glial sheaths [11]. The *Drosophila* SJ have received considerable attention because they share with the vertebrate tight junctions not only a common role but also several conserved components, suggesting that they could be homologue structures [11], [12]. In addition, there are also striking parallelisms at the functional and molecular level between the insect SJ and the vertebrate paranodal septate junctions [13], which are adhesion structures formed at the axon-Schwann cells contact areas on both sides of the nodes of Ranvier [14]. Thus, studying the *Drosophila* SJ is a way to identify new components of these multi-molecular adhesion complexes and to understand the general mechanisms controlling their assembly.

In this work we show that *cold* is specifically required for the organisation of the SJ in both epithelial tissues and in glial cells, where its activity is required for maintenance of the blood brain barrier. We present evidence suggesting that the *cold* product could behave as a membrane component of the septate junctions and we show that this gene, differing from *bou*, is required in a cell autonomous way.

## Results

### Cold is expressed in ectodermal derivatives and in a subset of glial cells

**Figure 1:**
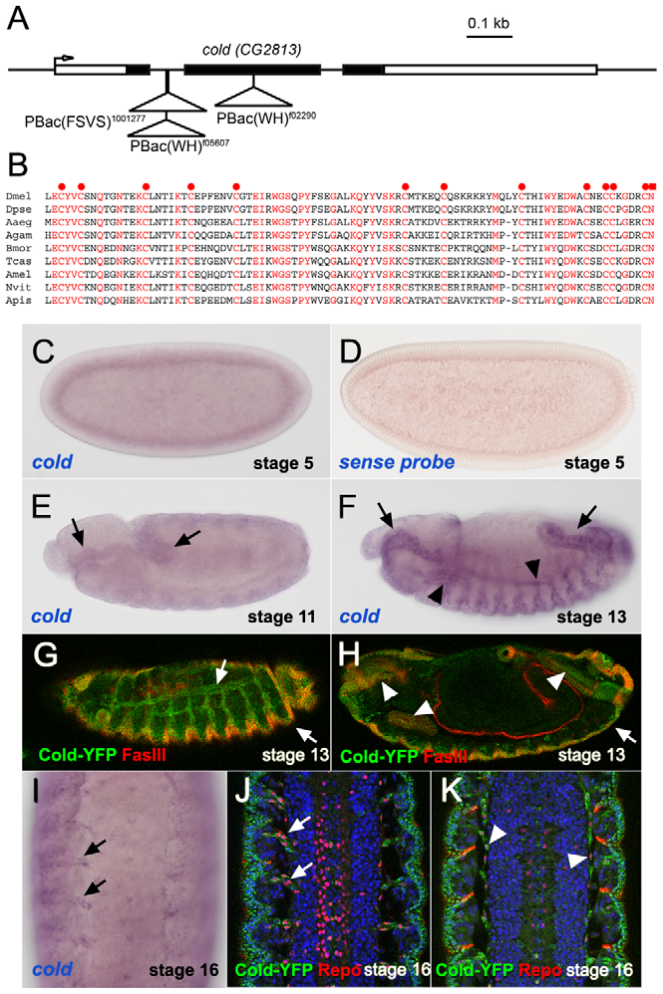
Structure and expression pattern of the *cold* gene. (A) *coiled/CG2813* genomic region, showing the localisation of the *cold PBac* insertions used in this work. (B). Protein sequence alignment corresponding to the Ly6 domain of different insect Cold orthologues. Invariant residues are shown in red. The 12 invariant cysteines and C-terminal asparagines, are marked respectively by red circles and a red square. See Materials and Methods for the species full names. (C,E,F) *In situ* hybridisation showing the *cold* mRNA distribution at different embryonic stages. Note at stage 5 an uniform signal corresponding to a maternal transcript and progressive accumulation of *cold* mRNA in the foregut and hindgut primordia at stage 11 (arrows), and in foregut, hindgut (arrows), trachea and salivary gland (arrowheads) of stage 13 embryos. (D) No signal was observed using a *cold* sense RNA probe. (G,H) Stage 13 *PBac^1001277^* embryos expressing a YFP-Cold fusion protein and stained for FasIII. The YFP signal was detected in the trachea and epidermis (white arrows) and in salivary gland, fore and hindgut (white arrowheads). (I) Ventral cord of a stage 16 embryo revealing *cold* transcript accumulation in cells associated with the nerve tracks (arrows). (J,K) Stage 16 embryos stained for Repo (red) and the YFP-Cold fusion protein (green), which is expressed in a subset of glial cells placed in the ventral cord surface (arrowheads) and associated with the exiting nerves (arrows).

To begin the functional characterisation of new members of the *Drosophila* Ly6 superfamily, we searched in public stock collections for potential mutants affecting their activity and focused in the analysis of the *CG2813* gene, for which three different putative mutants are available for genetic studies (see below). While we were preparing this manuscript, a study reported an analysis of *CG2813* mutants and named this locus as *coiled* (*cold*) [10]. Thus, thereafter we will refer to *CG2813* as to *coiled*. The *cold* gene codes for a single Ly6 domain, whose primary sequence appears to be well conserved among insects. In fact, a *cold* orthologue can be recognised in several available fully sequenced insect genomes (Fig. 1B and data not shown). This indicates that, in contrast with other Ly6 *Drosophila* paralogues, which are found exclusively in the drosophilid lineage [7], the *cold* product could be part of an ancient genetic network common to all insects. Genetic analysis of three independent mutant lines carrying PiggyBac insertions in different regions of the *cold* locus (Fig. 1A) revealed that all of them behave as recessive embryonic lethal alleles belonging to a single complementation group. These observations indicate that *cold* function is essential for embryonic development. Consistently, remobilisation of the *PBac^f05607^* transposon restored fly viability both in homozygosis and in heteroallelic combinations, suggesting that this insertion is responsible for the observed lethality.

To begin the analysis of *cold* function, we studied by *in situ* hybridisation the embryonic distribution of its transcript. In early embryos, we detected an uniform signal corresponding to a *cold* mRNA maternal contribution (Fig. 1C–D). After cellularisation, *cold* is expressed in the ectoderm and at low levels until stage 11, when its transcript begins to accumulate in the fore and hindgut primordia (Fig. 1E). By stage 13, *cold* is expressed at high levels in epithelial derivatives, including the tracheal network, the fore and hindgut and the salivary glands (Fig. 1F). We also detected a weak expression in the embryonic epidermis and, at late stages, in cells associated with the nerve tracks exiting the ventral cord (Fig. 1I). Furthermore, we monitored the expression of YFP (Yellow Fluorescent Protein) in embryos carrying the *PBac^1001277^* protein trap insertion, which is placed in the first *cold* intron and produces an in frame YFP fusion with the Cold protein (Fig. 1A). The expression pattern of this protein matches the observed distribution of the *cold* transcript, as we observed Cold-YFP in all epithelial derivatives by stage 13 (Fig. 1G–H). At later stages, this fusion protein was also detected in a subset of Repo-positive glial cells [15] seen both at the surface of the ventral cord and in close association with the nerve tracks (Fig. 1J–K). Thus, these observations indicate that *cold* could have a role not only in epidermal tissues but also in glial cells.

### Cold is required for tracheal morphogenesis and SJ organisation

**Figure 2:**
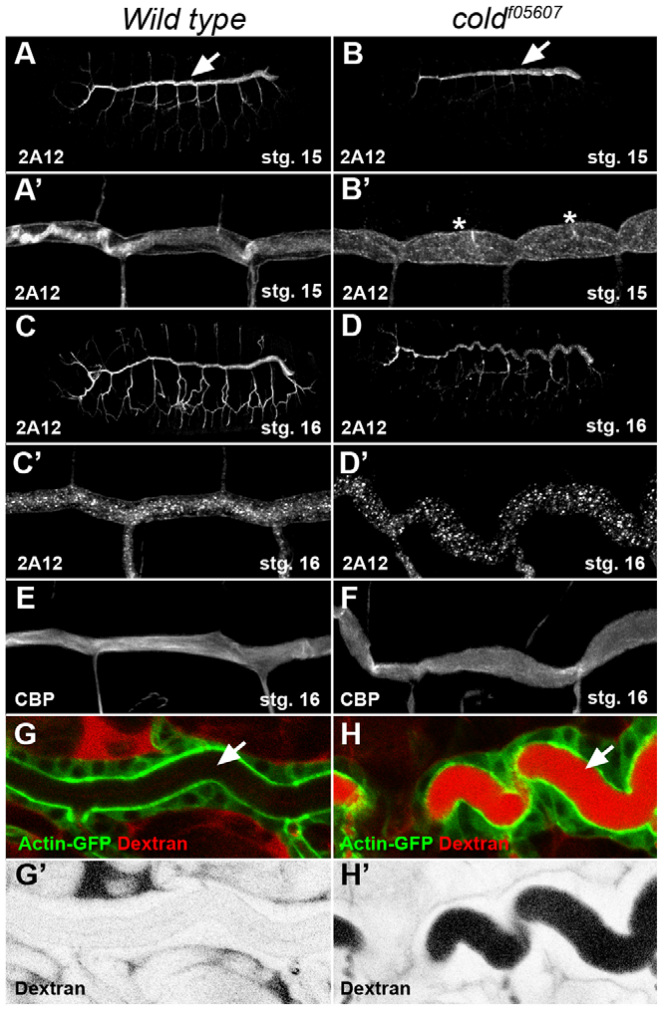
The tracheal morphology and paracellular barrier integrity are perturbed in *cold* embryos. (A–D) Projections of confocal stacks corresponding to wild type and *cold^f05607^* embryos staged as indicated and stained for the 2A12 tracheal luminal antigen. The same trachea are shown at higher magnification in panels A′–D′. At stage 15, the morphology of the tracheal dorsal trunk (arrows) is affected and displays a series of cysts (asterisks) visible in *cold* mutants. By stage 16, the dorsal trunk adopts a convoluted shape. (E–F) Projections of confocal stacks showing the tracheal dorsal trunk stained with fluorescent chitin binding probe (CBP). In the wild type, the chitin cable displayed a fibrous structure that was lost in *cold* embryos of the same stage. (G,H) Single confocal sections showing a view of the dorsal tracheal trunk marked by ActinGFP (green) and corresponding to stage 16 live embryos injected in the hemolymph with rhodamine 10 kDa dextran (red). (G′,H′) show a greyscale negative image of the red channel shown in G,H.

We examined embryos homozygous for the *cold^f05607^* insertion in search of visible phenotypes. For this, we focused on the development of the tracheal network, a tissue where *cold* is expressed at high levels. Staining of the embryonic tracheal system with the 2A12 luminal marker revealed that the overall organisation and branching pattern of this tubular network was preserved in *cold* mutants (Fig. 2C–D). However, both the shape and the length of the tracheal dorsal trunk segments were abnormal in this mutant (Fig. 2A–D′). Differing from the wild type, this structure appeared in stage 15 *cold* embryos as a succession of bulging cysts (Fig. 2A–B′) and, by stage 16, an abnormally convoluted tube (Fig. 2C–D′). Staining of the tracheal chitin cable with a fluorescent chitin binding probe (CBP) [16], showed that its fibrous structure was disorganised in the mutant embryos and chitin was deposited as an amorphous material in the tracheal lumen (Fig. 2E,F). These phenotypes are highly reminiscent of mutants affecting the formation of the septate junctions (SJ) [12], and prompted us to test whether the paracellular barrier preventing solute passage through epithelial layers is functional in *cold* embryos, a diagnostic character for mutants involved in SJ organisation [17]. We monitored the ability of a 10kDa fluorescent dextran dye injected into the body cavity to diffuse into the tracheal lumen of *cold* live embryos. In sharp contrast with the wild type controls, we observed a rapid diffusion (<30 minutes) of this soluble dye into the lumen of stage 16 *cold* mutant trachea (Fig. 2G–H′), showing that *cold* is essential in this tissue for organisation of a functional paracellular barrier.

**Figure 3:**
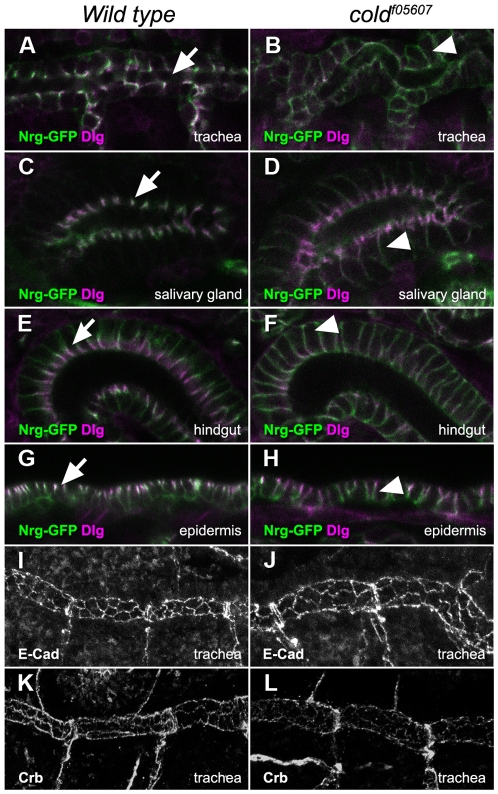
*cold* is required for the organisation of epithelial septate junctions. (A–H) Single confocal sections showing the subcellular localisation of the Nrg-GFP (green) and Dlg (magenta) proteins in different epithelial tissues belonging to stage 16 wild type and *cold^f05607^* embryos. In wild type embryos, Nrg-GFP and Dlg accumulated in the apical part of these epithelia (arrows). In the mutant embryos, the Nrg-GFP protein appeared uniformly distributed in the lateral part of the cells (arrowheads), whereas some apical accumulation of Dlg protein was still visible. (I–L) Projections of confocal stacks representing the trachea of wild type or *cold^f05607^* embryos, stained as indicated. E-cadherin and Crumbs localised to the apical part of the tracheal cells in both genetic backgrounds.

**Figure 4:**
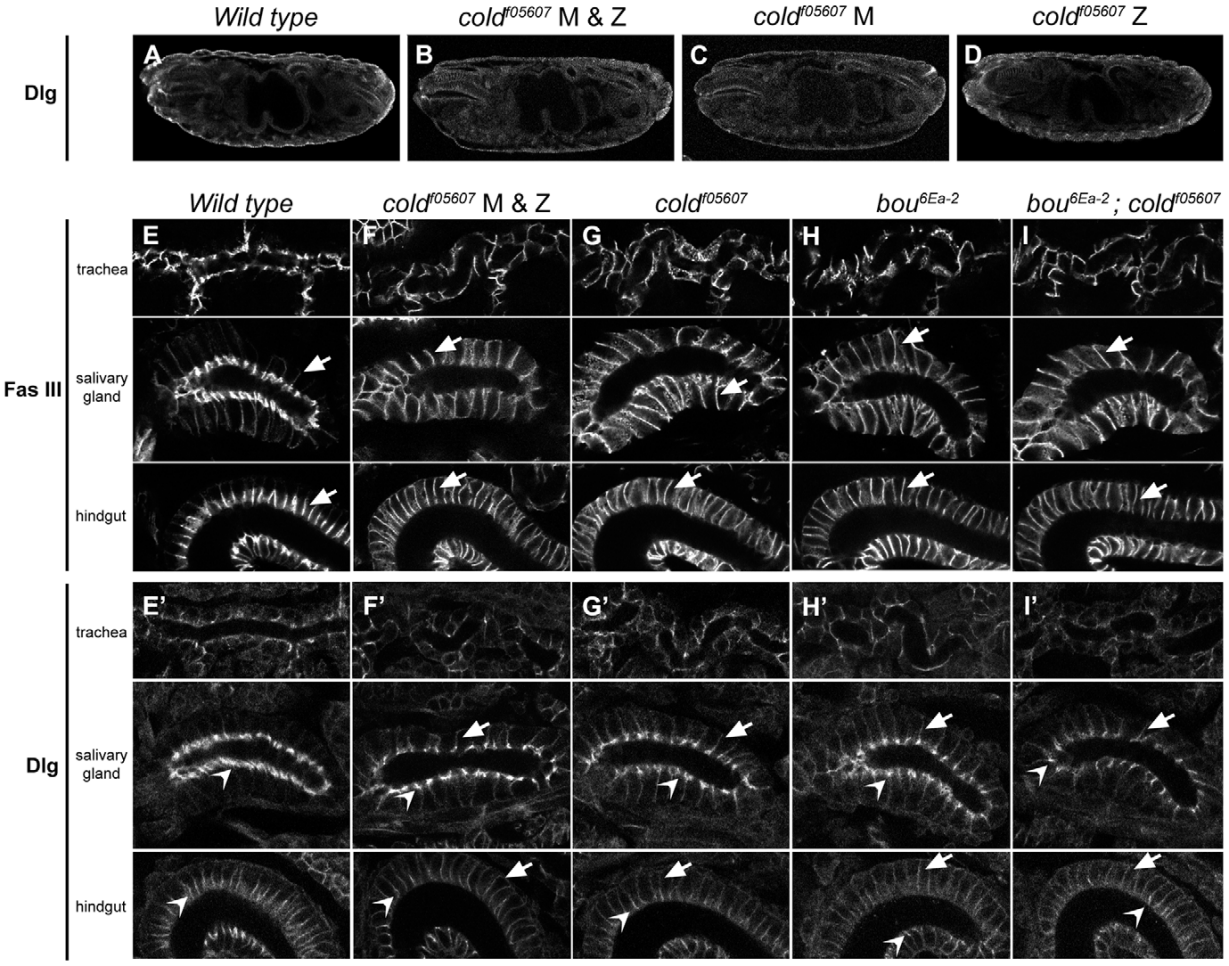
*cold* is not required for establishment of epithelial cell polarity. (A–D) Single confocal sections showing the overall morphology of either wild type embryos or mutant combinations lacking the *cold* maternal contribution (*cold* M), the zygotic one (*cold* Z) or both (*cold* M & Z), all stained for Dlg. (E–I′) Subcellular distribution of FasIII and Dlg in trachea, salivary gland and hindgut of stage 16 embryos of the indicated genotypes. Notice FasIII uniform distribution in the lateral membrane of the mutant tissues (E–I, arrows). A portion of Dlg (E′–I′) was redirected to the lateral membrane in the mutant tissues (arrows), although apical accumulation was still visible in the salivary gland and hindgut cells (open arrowheads).

We then studied in the same mutant background the subcellular distribution of Nrg-GFP [18] and Dlg [19], two SJ markers. The localisation of Nrg-GFP was perturbed in all epithelial tissues examined in *cold* mutant embryos (Fig. 3A–H). Instead of accumulating in the most apical part of the cells, Nrg-GFP appeared homogenously distributed along their lateral side in the trachea, the salivary glands, the hind-gut and the epidermis (Fig. 3A–H). The localisation of Dlg was affected in a similar way, but we observed that a portion of this protein was still accumulating in the apical part of the cells, suggesting that apico-basal polarity is not completely lost in the *cold* embryos (Figs. 3A–H and 4F′–G′). Consistently with this idea, the localisation of E-Cadherin and Crumbs, respectively apical junction and cell polarity markers [20], [21] was not altered in *cold* mutant trachea, suggesting that this gene is not required for cell polarity or assembly of other adhesion structures (Fig. 3I–L).

However, the *cold^f05607^* homozygous embryos could still contain some wild type product supplied maternally and capable of masking its requirements during early establishment of cell polarity, as observed with other genes required for SJ assembly such as *coracle*, *NrxIV*, *yurt* and the *Na^+^/K^+^ ATPase* [22]. To address this issue, we generated embryos in which the *cold* maternal contribution was missing, taking advantage of the FLP-DFS (FLP-recombinase-dominant female sterile) technique [23]. Embryos lacking the *cold* maternal contribution but rescued paternally with a wild type allele did not show any phenotype and survived into adulthood (Fig. 4C and data not shown). In contrast, mutant embryos lacking both maternal and zygotic contributions died during embryogenesis but did not display obvious morphological defects and were indistinguishable from embryos lacking only the *cold* zygotic contribution (Fig. 4A–D). Notably, they exhibited similar defects in SJ organisation, as revealed by staining with antibodies against FasIII (Fig. 4E–I) and Dlg, which was still seen accumulating in the most apical part of the salivary gland cells in both types of embryos (Fig. 4E′–I′). Since *boudin*, another member of the Ly6 gene superfamily, is also required for SJ organisation [7], we wondered whether some degree of genetic redundancy could exist between *cold* and *bou*. The overall morphology of embryos either double or single mutant for these genes was identical and the distributions of FasIII and Dlg were also indistinguishable in the three mutant backgrounds (Fig. 4G–I′). Thus *cold* and *bou* do not show redundant activities during embryogenesis.

Altogether, these results indicate that the tracheal morphological phenotypes and the defects observed in the paracellular barrier of *cold* mutant trachea are likely to result from a specific defect in the assembly or maintenance of the epithelial SJ.

### Cold is required for blood brain barrier organisation

**Figure 5:**
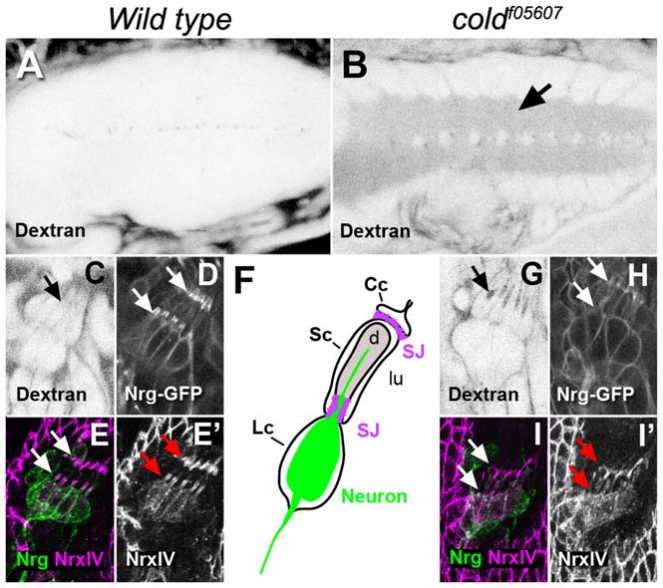
The *cold* gene is required for blood-brain barrier organisation. (A–B) Single confocal sections taken at the level of the ventral cord and showing in negative the distribution of 10 kDa rhodamine dextran injected into the body cavity of live stage 17 embryos. Notice dye penetration in the neuropile region of the *cold* mutant (arrow). (C,G) Confocal sections taken at the level of the lateral chordotonal organ of dextran injected stage 16 live embryos, showing dye accumulation in the lumen of these organs in the *cold* mutant (arrows). (D,H) Distribution of Nrg-GFP in the chordotonal organ of stage 16 live embryos, showing accumulation of this protein in the junctions existing between cap and scolopal cells (arrows) and ligament and scolopal cells (arrowheads). Notice that Nrg-GFP accumulation was lost in the mutant embryos. (E,I) Projections of confocal stacks showing the distribution of NrxIV (magenta) and the BP104 antibody-reactive Nrg neural isoform (green) in the lateral chordotonal organs of stage 17 embryos. (E′,I′) correspond to the magenta channel shown in E,I. The NrxIV SJ marker was not properly localised at the level of the cell junctions in the mutant embryos (white and red arrows). F. Cartoon representing the cellular composition of one single scolopal unit. Cc, cap cell; Sc, scolopal cell; Lc, ligament cell; d, dendrite; lu, lumen; SJ, septate junctions.

Insect pleated SJ are not exclusive of epithelial cells and are also seen at the cell contacts existing between certain types of glial cells [24]. In the embryonic ventral cord, the presence of SJ in the subperineural glia is essential for the formation of the so called blood-brain barrier, a physiological fence essential for brain insulation from the hemolymph [25]. Given that *cold* is expressed in surface glial cells, we monitored if dye injected into the hemolymph could penetrate into the ventral cord of *cold* mutants 22 hours old, when the blood-brain barrier is fully established [26]. Our results show that a 10 kDa dextran dye readily diffused into the ventral cord of *cold* mutant embryos, whereas it was efficiently excluded from this structure in wild type embryos of the same age (Fig. 5A–B). We also analysed dye diffusion in the peripheral nervous system lateral chordotonal organs, which are insulated from the hemolymph by a specific set of glial cells [27], [28]. We observed that the injected dye diffuses into the lumen of the chordotonal organs in stage 17 *cold* embryos (Fig. 5G), whereas SJs established between the cap, scolopal and ligament cells (Fig. 5F) [28] prevented dye intake in the wild type controls (Fig. 5C). In fact, the SJ markers Nrg-GFP and NrxIV were not seen accumulating at the contact regions between these cells in a *cold* mutant background (Fig. 5D–E′ and H–I′). These observations demonstrate that maintenance of an efficient paracellular barrier and proper distribution of SJ markers depends on the activity of *cold* in the nervous system.

### The *cold* gene is autonomously required for SJ organisation

**Figure 6:**
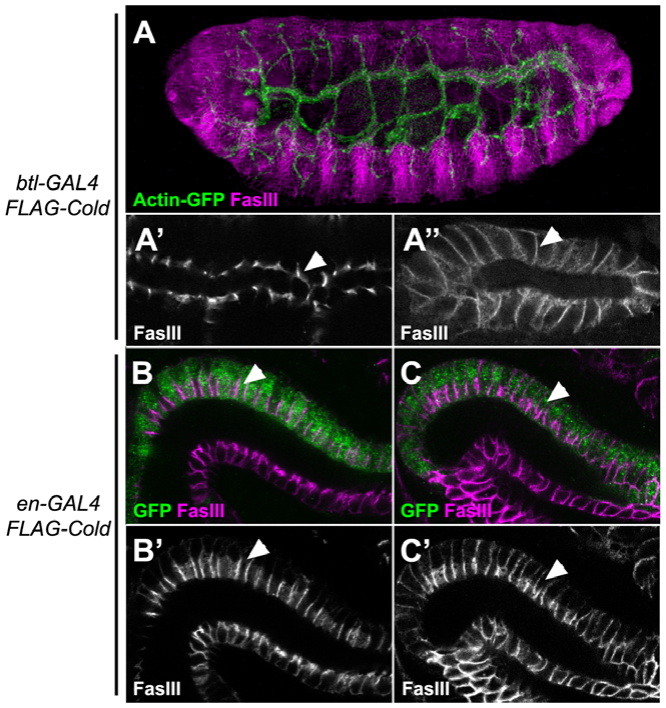
The embryonic *cold* SJ phenotypes are cell autonomous. (A) Projection of a confocal stack showing a stage 16 *cold^f05607^* homozygous embryo stained for FasIII (magenta) and expressing FLAG-Cold and Actin-GFP (green) proteins under the control of *btlGAL4*. (A′,A″) Single confocal sections at a higher magnification of the trachea dorsal trunk (A′) and the salivary gland (A″) of the same embryo, showing the distribution of the SJ marker FasIII. Notice the delocalisation of this marker in the salivary gland (arrowhead) and its normal distribution in the trachea (arrowhead). (B–C′) Distribution of FasIII (shown in magenta in B,C and in greyscale in B′,C′) in the hindgut of stage 16 embryos expressing FLAG-Cold in the *engrailedGAL4* territory, marked by GFP (green). In wild type embryos, expression of FLAG-Cold in the *en* cells (green) did not affect the localisation of the FasIII marker in the apical portion of the lateral membrane (arrowheads) (B,B′). In *cold^f05607^* homozygous embryos normal apical accumulation of FasIII was only observed in the *en* cells (green, arrowheads).

**Figure 7:**
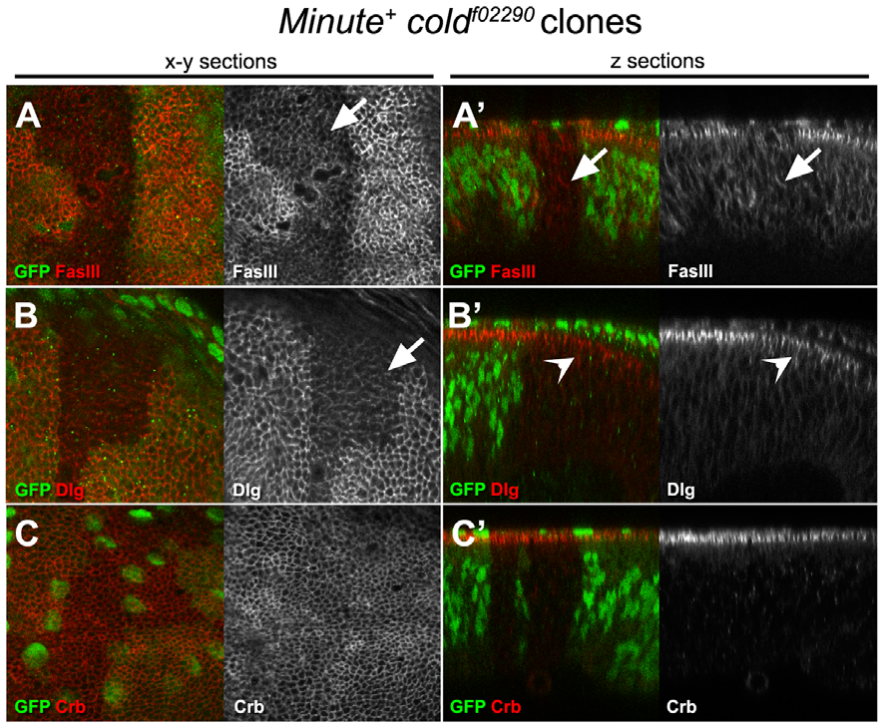
The *cold* gene is autonomously required in the wing disc for SJ organisation. (A–C′) Confocal pictures of third larval instar wing imaginal discs containing *M^+^ cold^f02290^* clones generated in first instar larvae and stained as indicated. The homozygous *cold* mutant cells lacking GFP (green) can be distinguished from the surrounding GFP positive heterozygous tissue. A–C correspond to optical planar x-y sections taken at the level of the SJ and A′–C′ show optical z-sections of the same discs. The planar views revealed abnormally low levels of FasIII and Dlg (red in left panels, greyscale in right panels) in the apical part of the *cold* mutant cells (arrows). The z planes show that Dlg was found accumulating in a wider apical domain (open arrowhead), whereas FasIII was distributed uniformly along the lateral part of the cells (arrow). A normal Crb apical distribution (red in left panel, greyscale in the right panel) was observed in *cold* mutant cells.

Previous analysis of the *bou* Ly6 gene, which is also required for SJ formation, indicates that the mutant phenotypes for this gene are not cell autonomous, as presence of wild type neighbour cells can rescue the SJ defects seen in *bou* mosaic embryos [7]. To test whether this feature also applies to the *cold* phenotypes, we directed expression of a FLAG tagged form of Cold in the tracheal cells of a *cold* mutant embryo, using the *btlGAL4* driver. The SJ marker FasIII delocalisation phenotype observed in *cold* trachea was fully rescued by FLAG-Cold expression (Fig. 6A–A′). This indicates that this protein is fully functional and further confirms that the *cold* gene is responsible for the observed tracheal phenotypes. However, unlike the Bou protein, targeted Cold expression in the trachea did not restore proper FasIII localisation in other tissues, like the salivary gland (Fig. 6A″). We further confirmed the full autonomy of the *cold* requirements by monitoring FasIII distribution in the hindgut of mutant embryos expressing FLAG-Cold under the control of the *engrailedGAL4* driver, which is only expressed in the dorsal half of this epithelial tube [29]. In fact, FasIII appeared correctly localised only in the cells expressing the FLAG-Cold protein (Fig. 6C–C″). Thus, our results show that *cold* rescuing activity is neither able to diffuse from tissue to tissue (like in the case of *bou*) nor between neighbour cells belonging to the same epithelium. In line with these results, we found that *cold* is also autonomously required for proper SJ maintenance in the epithelial cells that form the imaginal discs. Indeed, in mosaic third larval instar wing discs containing large *cold^f02290^ Minute^+^* clones, we observed that the *cold* mutant cells fail to accumulate the FasIII marker in their most apical part, unlike the surrounding wild type cells (Fig. 7A,A′). Consistently with the idea that *cold* is not involved in cell polarity maintenance, we also observed that a small amount of Dlg and normal levels of Crumbs are present in the most apical part of the mutant cells (Fig. 7B–C′).

### Cold is present at the membrane and stably associates with regions containing SJ

**Figure 8:**
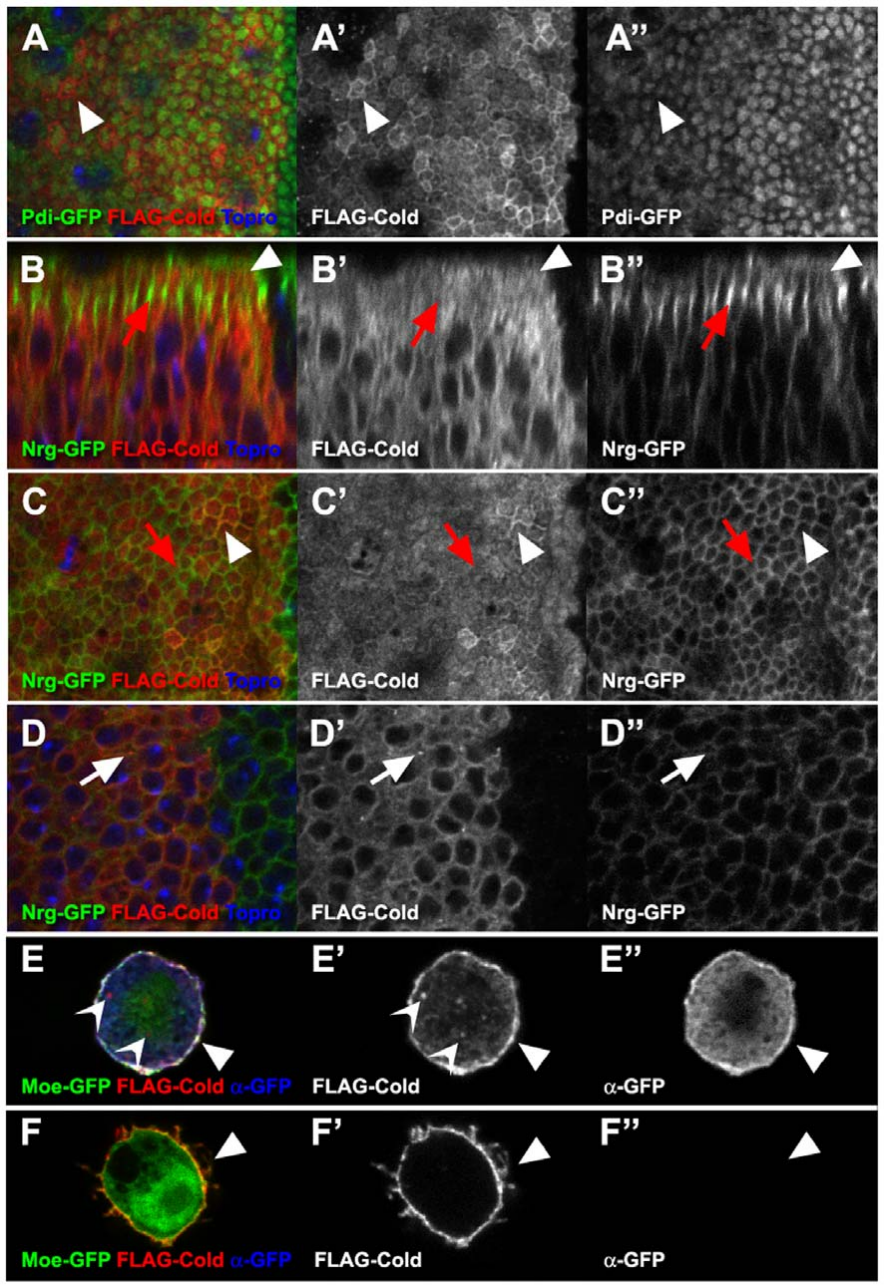
FLAG-Cold subcellular localisation in the wing epithelium and in S2 cells. (A–D″) Confocal images of third larval instar wing discs expressing the FLAG-Cold protein (shown in red in left panels, greyscale in the mid panels) in the *apGAL4* domain. The A–A″ and C–C″ show x-y planar views of the apical part of the epithelium, whereas D–D″ shows a more basal region and B–B″ a z-section. Accumulation of FLAG-Cold was seen in a cell apical region containing weak levels of Nrg-GFP (B–B″ and C–C″ white arrowheads) and no Pdi-GFP (A–A″, white arrowheads). Regions containing high levels of Nrg-GFP and corresponding to the SJ did not show FLAG-Cold accumulation (B–B″ and C–C″, red arrows). FLAG-Cold was also seen in internal vesicles (D–D″, white arrows). E–F″ Confocal images of S2 cells expressing FLAG-Cold (red in left panel, greyscale in the mid panel) and Moesin-GFP (GFP fluorescence shown in green, left panel). In permeabilised cells (E–E″), both FLAG-Cold and Moesin-GFP were detected by antibodies in the cell interior and at the membrane (anti-GFP shown in blue, left panel and in greyscale, right panel). In these conditions, we also observed internal vesicles accumulating FLAG-Cold (open arrowheads). In non permeabilised cells (F–F″), FLAG-Cold was detected at the membrane (arrowhead), whereas the cell interior was not accessible to the antibodies.

Lacking specific antibodies against Cold, we took advantage of our FLAG-Cold fusion protein to analyse its subcellular distribution in wing disc epithelial cells, using *apterousGAL4* as a driver. The FLAG-Cold was detected inside the cells, where we observed extensive co-localisation with the endoplasmic reticulum marker Pdi-GFP [30] (Fig. 8A–A″). We also detected a slight concentration of FLAG-Cold in the most apical part of the cells, in a region free of endoplasmic reticulum that could correspond to the plasma membrane, as it contained low levels of Nrg-GFP (Fig. 8B–C″). Yet, the FLAG-Cold protein did not obviously accumulate in the membrane regions displaying the highest levels of Nrg-GFP and harbouring the SJ (Fig. 8B–C″). In addition, we observed in more basal regions internal vesicles containing FLAG-Cold which do not stain positively for Nrg-GFP (Fig. 8D–D″). Thus, although FLAG-Cold is not preferentially associated with the SJ, our observations indicate that part of this protein could be present in the plasma membrane, as it is the case in S2 cultured cells. In fact, FLAG-Cold is readily detected at the cell surface of non permeabilised S2 cells that were stained in conditions that prevent antibody access to the interior of the cell (Fig. 8F–F″). In permeabilised cells, we also observed presence of FLAG-Cold in internal vesicles, as previously noticed (Fig. 8E–E″) [10].

**Figure 9:**
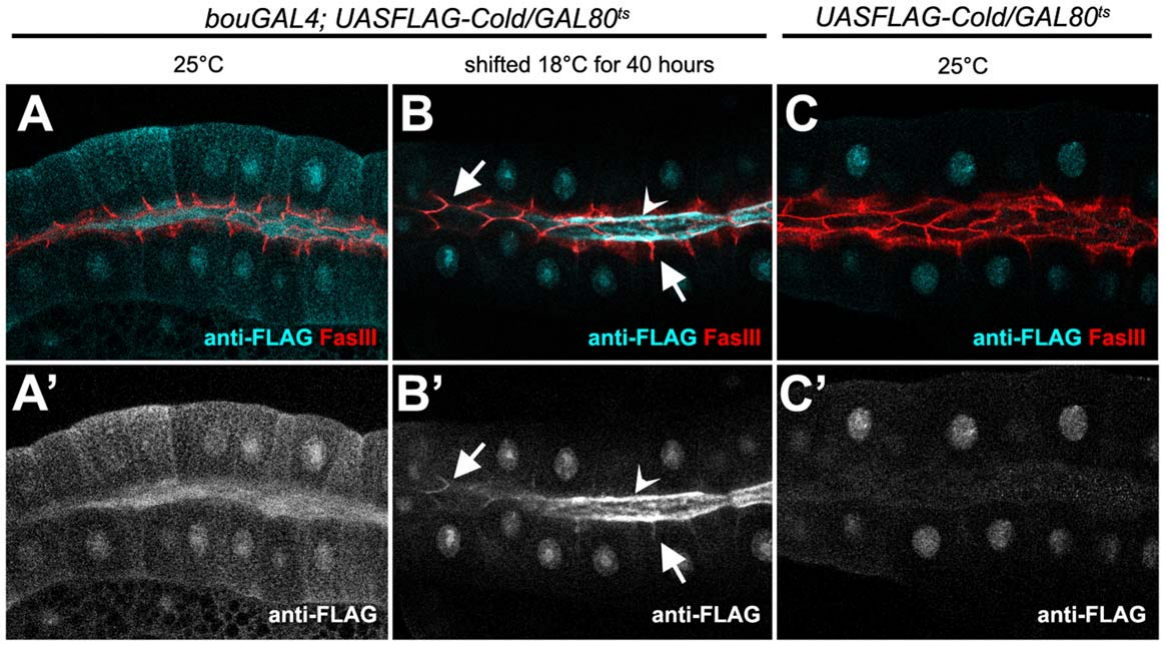
FLAG-Cold is stably associated with the SJ in larval salivary glands. (A–C′) Confocal single sections of salivary glands belonging to third instar larvae carrying the *UASFLAG-cold* construct and the *tubulinGAL80^ts^* repressor. (A,A′) At 25°C and in presence of the *bouGAL4* driver, FLAG-Cold (cyan in upper panels, greyscale in lower panels) was present at high levels in the salivary gland lumen and the cell body. (B,B′) After 40 hours at 18°C the cell body staining disappeared but FLAG-Cold persisted in the salivary gland lumen (open arrowhead) and in lateral cell regions (arrows) accumulating FasIII (seen in red). A nuclear staining was also observed but it corresponds to a unspecific interaction of the FLAG antibody, as it was also present in larvae not carrying the *bouGAL4* driver (C,C′).

These observations are consistent with the idea that FLAG-Cold could be a membrane protein cycling between internal compartments and the plasma membrane, but we reasoned that the high levels of FLAG-Cold produced in these experiments could saturate the cell, obscuring its potential accumulation in particular subcellular compartments. To analyse the localisation of this protein in a less saturated background, we took advantage of the GAL80^ts^ repressor to switch down FLAG-Cold production during development [31]. Accordingly, we expressed this protein in presence of the *tubGAL80^ts^* repressor in third larval instar salivary glands, using *bouGAL4* as a driver [7]. In larvae growing at 25°C, we detected high levels of FLAG-Cold in the lumen and in the cell bodies of the salivary gland cells, but no preferential accumulation at the SJ level (Fig. 9A,A′). Then, we switched-off FLAG-Cold expression by shifting the fly cultures to 18°C 40 hours prior to dissection. In these conditions, the FLAG-Cold protein was still seen in the salivary gland lumen, but could also be detected at low levels in a lateral cell region containing the FasIII SJ marker (Fig. 9B,B′). This weak staining was not observed in control glands lacking the *bouGAL4* driver (Fig. 9C,C′), indicating that upon expression, a small portion of FLAG-Cold seems stably associated with SJ-containing regions.

## Discussion

### Is *cold* specifically required for SJ organisation?

The profusion of *Drosophila* Ly6 paralogues (45 members) and the variety of their expression patterns [7] suggest that mutants for these genes could *a priori* display the most various phenotypes, as it is the case for the three fly Ly6 genes characterised in so far: *rtv* [8], [16], *sss* [9], [32] and *bou* [7]. However, a recent report pointed out that three other *Drosophila* Ly6 proteins, Coiled, Crooked and Crimpled participate in a non redundant way in the same process as Bou: the organisation of epithelial septate junctions [10]. Our genetic characterisation of *cold* mutants further confirms that this gene is required for SJ organisation in epithelial tissues, and shows by direct comparison with *bou* mutants that both elicit undistinguishable phenotypes. Still, besides their diagnostic set of 10 cysteines, the primary sequences of Bou, Coiled, Crooked and Crimpled are remarkably different, making impossible to predict a common molecular role. Given their structural divergences and the versatility of the Ly6 domain, they could in principle bind to different molecular partners. Our analysis of *bou cold* double mutant embryos indicates that at least these two genes do not exert redundant functions during SJ assembly and cell polarity establishment. Thus, the available data are coherent with the idea that these proteins have non-exchangeable roles, despite their similarities at the phenotypic level. Interestingly, the four Ly6 genes implicated in the organisation of the fly SJ have highly conserved orthologues in other insects, such as the honey-bee [7], pleading for a hardwired role of these proteins in SJ assembly.

Our analysis of embryos lacking both the *cold* zygotic and maternal contributions indicates that this gene is unlikely to have a role during the establishment of cell polarity. Thus, while some SJ components such as Yurt, NrxIV, Coracle and the Na^+^/K^+^ ATPase are also necessary for this process [22], the activity of Cold seems dispensable. Hence, it seems that this protein would participate in a genetic module whose role is solely required for the assembly of SJ. It will be interesting to test whether this also applies to the three other Ly6 genes affecting SJ organisation, as a differential requirement could provide hints facilitating the recognition of their specific partners.

A recent genetic screen identified *cold* as a gene required in the embryonic epidermis for efficient reestablishment of epithelial integrity upon injury [33]. This observation indicates that the integrity of the whole SJ adhesion complex could be required for wound healing or, alternatively, that *cold* may have a specific role in this process. Further analysis of the role of the SJ adhesion structures during epithelial repair will allow to clarify this issue. In any case, this observation shows that upcoming functional analysis of the *Drosophila* Ly6 proteins is likely to contribute to a better understanding of many developmental and physiological processes in which this versatile module has been co-opted.

### Is Cold a SJ component?

Previous studies in S2 cells pointed out that a Cold tagged version expressed in these cells accumulates in endocytic vesicles [10]. We have further analysed the localisation of a functional FLAG-Cold fusion protein, both in S2 cells and in developing tissues containing SJ. Our findings are consistent with the idea that Cold, predicted to be GPI anchored by the bigPI software [34], is associated not only with the endoplasmic reticulum and internal vesicles but also with the plasma membrane. In the salivary glands, we observed that a small amount of FLAG-Cold was stably associated with SJ containing regions, as if making part of a complex localising to this membrane compartment. Interestingly, a similar accumulation has been observed with a HA-Bou tagged version in the same tissue [7]. It is thus possible that a small subset of both proteins could contribute to the organisation of the SJ by interacting with each other and/or with other SJ components. However, it is premature to conclude that Cold function is circumscribed to the lateral membrane region containing the SJ, as other valid alternatives exist. In fact, the FLAG-Cold protein was also seen in other subcellular compartments, and it is difficult to ascertain where it exerts its primary activity. For instance, it has been shown that the SJ component NrxIV is re-localised to internal vesicles in *cold* mutant embryos [10]. Although this phenotype is also observed in embryos lacking known SJ components such as Coracle and Nrv2 [10], the Ly6 proteins could indeed play a role in the vesicular trafficking of these proteins or in their preassembly into larger macro-complexes en route to the membrane. As the intracellular traffic seems to play a key role in the early assembly of the SJ [35], it will be interesting to compare the paths followed by Ly6 proteins and known SJ components during the formation of these structures. Concerning the traffic of the Ly6 proteins themselves, it is clear that Cold differs from Bou in two related aspects: it behaves in a cell autonomous way and we have not found any evidence indicating that this protein could travel from cell to cell. Thus, although these proteins may meet at the level of the SJ or in other subcellular compartments, these observations implicate that they do not always traffic together. It will be interesting to analyse whether this differential behaviour provides a rationale for their non-exchangeable roles.

### A conserved role for Cold in the nervous system?

Our results show that *cold* is expressed in a subset of glial cells and is required for organisation of the blood-brain-barrier in the *Drosophila* nervous system. Thus, it is possible that vertebrate members of the Ly6 superfamily could fulfil an analogous role in the formation of the paranodal junctions existing in the contact areas between axons and Schwann cells [14]. The high variability observed in the Ly6 domains primary sequence precludes identification of vertebrate orthologues corresponding to the *Drosophila* genes. However, the genetic networks in which these proteins are implicated could be better conserved, as insect SJ and paranodal junctions share a significant number of components [36]. Thus, future functional studies in *Drosophila* and vertebrates may reveal analogous roles for apparently unrelated Ly6 proteins, as it is the case for the four *Drosophila* Ly6 members participating in SJ assembly.

## Materials and Methods

### Sequence analysis

We used the TBLASTN program to search for *cold* orthologues in insect genome databases using the BLAST search program [37] and the ClustalW program to create the alignments. The species considered are Dmel, *Drosophila melanogaster*; Dpse, *Drosophila pseudobscura*; Aaeg, *Aedes aegypty*; Agam, *Anopheles gambiae* (Diptera). Bmor; *Bombyx mori* (Lepidoptera). Tcas, *Tribolium castaneum* (Coleoptera). Amel, *Apis mellifera*; Nvit, *Nasonia vitripennis* (Hymenoptera). Apis, *Acyrthosiphon pisum*, (Hemiptera).

### Genetics

Full definitions of the stocks used can be found in Flybase [38], and include the *cold* alleles *P(WH)Bac^f05607^* and *P(WH)Bac^f02290^* and the strains *P(GawB)bou^PG27^*, *bou^6ea-2^*, *P(PTT-GA)Nrg^G00305^*, *P(PTT-un1)Pdi^74-1^*, *P(GawB)ap^md544^*, *btlGAL4 UASActinGFP*, *P(en2.4-GAL4)^e16E^ UASGFP* and *tubGAL80^ts^*. *The PBac(FSVS-1)^1001277^* insertion was generated by the CPTI (http://www.flyprot.org/). The *FM7c-ActinLacZ, CyO-wglacZ* and *CyO KrGAL4UASGFP* balancers were used for embryo genotyping. All experiments were carried out at 25°C, except the temperature shifts at 18°C, which where done 40 hours before dissection in cultures containing third larval instars of the *bou^PG27^/+; UASFLAG-Coiled/+; tubGAL80^ts^/+* genotype. The *cold* embryos lacking both the maternal and the zygotic contributions were recovered in the progeny of *hsFLP/+; P(ovoD1-18)^2La^ P(ovoD1-18)^2Lb^ FRT40A/cold^f05607^ FRT40A* females heat shocked two times for 1 hour at 37°C during larval stages and mated to *cold^f05607^/CyO-wglacZ* males. The somatic *Minute^+^ cold* clones were induced 48 hours after egg laying by 1 hour heat shock at 37°C in *w hsFLP*; *cold^f02290^ FRT40/M(2)24F^1^ ubiGFP FRT40A* larvae.

### Dye injection

Dye diffusion into trachea and chordotonal organs was analysed by injecting 10 mg/ml 10 kDa rhodamine-Dextran (Molecular Probes) with a micromanipulator into the body cavity of dechorionated stage 16 live embryos [17]. Diffusion into the nerve cord was studied in 22 hours old embryos. Samples were visualised within 20–30 minutes after injection with a Leica SP2 confocal microscope.

### Molecular biology

The FLAG tag coding sequence DYKDDDDK, flanked in each side by one A residue was introduced in frame by PCR within the Coiled coding region after the E25 residue, using specifically designed oligonucleotides and the LD16147 (DRGC) *coiled* cDNA as template. The construct was then sequenced and subcloned into pUAST [39] for generation of transgenic flies or pAc5.1 (Invitrogen), for cell transfections.

### Cell culture

Cell culture, transfections and antibody stainings were carried out as in Koh et al. [32]. S2 cells co-transfected with pAcDMoe-GFP (kind gift from F. Payre, CBD, Toulouse, France) and pAcFLAG-Cold were fixed and stained in either permeabilising (PBS, 0.1% Triton-X100) or non-permeabilising (PBS) conditions.

### Immunohistochemistry

*In situ* hybridisation with clone LD16147 sense and antisense riboprobes were performed according to [40]. Embryos and larval tissues were fixed for 20–30 minutes in PBS 4% paraformaldehyde. Blocking, washings and over night incubation with primary and secondary antibodies was carried out in 0.1% Triton-X100 0.1% BSA. Primary antibodies included mouse anti-ßGal 1/100 (Promega), rabbit anti-ßGal 1/1000 (Cappel), mouse anti-FLAG 1/200 (Covance), rabbit anti-FLAG 1/100 (Sigma) rabbit anti-GFP 1/500 (Torrey), anti-NrxIV 1/100 (kind gift of H. Bellen), rat anti-Crb 1/500 (kind gift of U. Tepass), and monoclonals anti-2A12 1/10, 4F3 anti-Dlg 1/100, DCAD2 anti-DECad 1/20, BP104 anti-Nrg 1/100, 7G10 anti-FasIII 1/30, all from DSHB. Secondary FITC and TRITC conjugated antibodies and streptavidin were diluted 1/200 (Molecular Probes). We also used CBP-FITC 1/100 (NEB). Samples were mounted in Vectashield (Vector) and visualised with a LeicaSP2 confocal microscope.
